# Acoustic rhinometry: anatomic correlation of the first two notches found in the nasal echogram

**DOI:** 10.1016/S1808-8694(15)31303-3

**Published:** 2015-10-20

**Authors:** Carlos Eduardo Nazareth Nigro, Josiane Faria de Aguiar Nigro, Richard Louis Voegels, Olavo Mion, João Ferreira Mello Junior

**Affiliations:** ^1^ Ph.D. in Otorhinolaryngology, Supporting Professor, Medical School, UNITAU; ^2^ Ph.D. in Otorhinolaryngology, Supporting Professor, Discipline of Otorhinolaryngology, UNITAU; ^3^ Ph.D., Associate Professor, Discipline of Otorhinolaryngology, HCFMUSP; ^4^ Ph.D., Assistant Professor, Discipline of Otorhinolaryngology, HCFMUSP; ^5^ Ph.D., Assistant Professor, Clinical Otorhinolaryngology, HCFMUSP

**Keywords:** acoustic rhinometry, nasal valve, nose

## Abstract

The graphic obtained by acoustic rhinometry in Caucasian adult individuals with no nasal affections clearly shows two notches at the beginning of the nasal echogram. However, there are controversies in the literature concerning their anatomic correlation.

**Aim:**

The aim of this study was to obtain data that would contribute to the anatomic correlation of these two notches.

**Study design:**

Clinical prospective.

**Material and Method:**

We analyzed the nasal echogram of 35 volunteers in basal conditions, after decongestion and after obstruction of the nasal valve by using cotton impregnated with Vaseline.

**Results:**

We identified statistically significant reduction and increase of the cross-sectional area only for the second notch after obstruction of the nasal valve and after decongestion, respectively.

**Conclusion:**

The analysis of the results suggested that the first notch of the nasal echogram refers to the nostril and the second notch refers to the nasal valve as a whole.

## INTRODUCTION

The anterior portion of the nasal cavity, from the nostril to the nasal valve, is the point of maximum resistance to airflow^1^, and it is where the narrowest portions of the nasal fossa is located^2^, which is extremely important for nasal physiology. Acoustic rhinometry (AR) is more accurate for measurements of area and volume within the first 5 cm of nasal cavity, particularly effective in regions anterior to the narrowing areas, of up to 2.4cm from the nostril^3^; therefore, it is more applicable exactly in the most anterior portion of the nasal cavity^4^.

**Figure 1:**
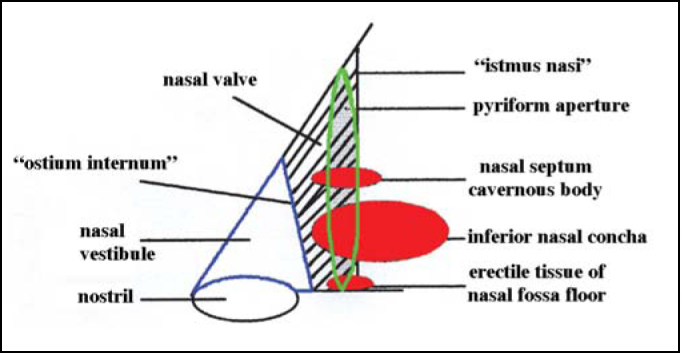
Diagram of anterior nasal portion.

To correlate anatomical structures of the anterior portion of the nose with rhinogram we have to clearly understand these structures (Figure 1). At the base of the nasal pyramid, we can locate anterior openings of right and left nasal fossae, named nostrils. They are laterally delimited by nose alas, right and left, and medially, by the columella. Columella corresponds to the mobile part of the nasal septum and it is an important structure to determine naso-labial angle. The nostrils have an oval gross format in Caucasians, its main axis is vertical, and in Black-descendents its longest axis is horizontal; in other races, its longest axis is oblique, having a rounded shape. Nasal vestibule is the opening to the nose. It belongs to nasal fossa, but it is distinguished from the rest of the nasal fossa because a large part of its internal lining is made of skin. It presents as a medial wall formed by septal cartilage and columella, which is formed by the junction between medial portion of inferior alar cartilage and its contralateral homologous. The lateral wall is concave and corresponds to the internal aspect of the lateral portion of inferior alar cartilage, which is recovered by skin with cilia and microcilia.

Posteriorly, we can find an orifice named ostium internum, which practically corresponds to the transition line between cutaneous squamous epithelium and pituitary mucosa, and it is the anterior segment of the region of nasal valve (NV). NV (Figure 2A) is anteriorly marked by the ostium internum, an orifice whose pear-shaped format, visualized under anterior rhinoscopy, is laterally limited by the inferior border of lateral superior cartilage, medially to the nasal septum and inferiorly to the floor of the nasal cavity, located 1 to 1.5cm from the nostril^5^. NV encompasses more posteriorly the pyriform orifice, floor of the nasal fossa, which presents erectile tissue on the region^6^, nasal septum cavernous body, and head of inferior nasal concha, which goes through the pyriform orifice (0.3cm in normal subjects, and after decongestion with nasal topical vasoconstrictor, it comes to its limit). These structures form the second segment of NV are called isthmus nasi and are located 1.65 to 2.65 cm from the nostril^5^. The inferior portion of superior lateral cartilage, at the ostium internum and the erectile tissue, at isthmus nasal, ensure mobility of these structures.

**Figure 2A:**
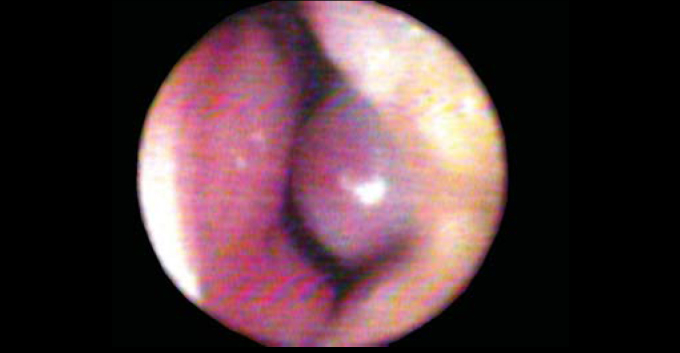
Photography of nasal valve in baseline conditions.

The graph traced by AR in adult normal Caucasian subjects clearly shows two notches at the beginning of the rhinogram. However, in the literature, we find disagreeing opinions about their anatomical correlation. The area of transversal section of the first notch (AST1) would be the nasal valve^7^, “isthmus nasi”^6^, ^8^, ^9^, “ostium internum”^10^, an area close to NV^11^, the junction of the nasal adapter and the nostril^12^ or the junction of nasal adapter and “isthmus nasi”^13^. The area of transversal section of the second notch (AST2) would refer to the head of the inferior nasal concha (CNI)^7^, ^8^, ^9^, ^10^, ^14^, ^15^ or to NV^12^, ^13^. The purpose of the present study was to define anatomical correlation between these two notches in adult Caucasian subjects without nasal affections.

## MATERIAL AND METHOD

We included in the present prospective study 35 adult Caucasian subjects without nasal affections (M:F = 18:17, mean age = 27.7 years). Exclusion criteria were: history of previous nasal or palate surgery; upper respiratory airway infections or rhinitis episodes; use of systemic decongestants, vasoconstrictors (VC), anti-histaminic, anti-cholinergic or nasal topical or systemic corticosteroids for the past 30 days. The study was approved by the Research Project Ethics Committee, HCFMUSP. The equipment used was Eccovision Acoustic Rhinometry System - Hood Laboratories Pembroke. The measurements were made according to recommendations of the Acoustic Rhinometry Standardization Committee.^16^

We conducted AR in the left nasal cavity of all subjects. We obtained 5 rhinograms in each subject to minimize variations between measurements, in each of the three steps; however, since reproducibility of the exam is high^17^, we chose the graph that contained mean values of AST 1 and 2 for the analysis of the following conditions: 1) baseline conditions (Figure 2A); 2) after obliteration of the valve region opening with cotton soaked in liquid Vaseline (Figure 2B); 3) after 15 min of application of 3 graded jets of vasoconstriction (VC) with oxymetazoline hydrochloride at 0.05% (0.07 mg/jet) (Figure 2C). Graphs were analyzed to measure AST and distance up to the lowest notch point (DAST) for both notches, which were calculated by the computer. We measured the distance from the beginning of notches, measuring the first descending point in both notches.

**Figure 2B:**
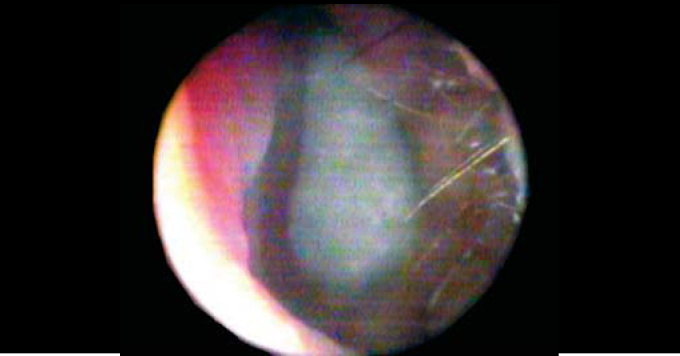
Photography of opening of nasal valve occluded by cotton ball soaked in liquid Vaseline.

**Figure 2C:**
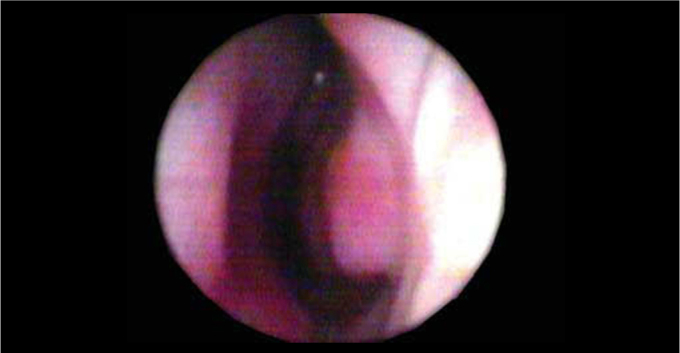
Photography of nasal valve after nasal topical vasoconstrictor.

Data were statistically analyzed by comparing the values obtained in both notches for the 3 rhinograms. The statistical test used was Analysis of Variance with repetitive measurements. Significant comparisons were made for p < 0.05, and we also applied Tukey multiple comparisons.

## RESULTS

Figure 3 represents the rhinogram observed in 3 conditions: baseline condition, with occlusion of NV, and after VC. Figures 4 and 5 presented respectively AST 1 and 2 observed in baseline conditions, with occlusion of NV and after VC in 35 studied volunteers. Table 1 refers to measures of AST1, AST2 and Table 2 refers to measurements of DAST1 and DAST2 measured in the 3 situations, as described above. Table 1 shows that there was no statistically significant variation of AST1 in the 3 situations. Comparing it with baseline condition, there was reduction and increase that were statistically significant for AST2 after occlusion of NV and after VC, respectively. Table 2 shows that there were no statistically significant differences for DAST1 in the 3 situations and after occlusion of NV there was statistically significant increase of DAST2 when compared to baseline condition. Even though DAST2 post-VC was reduced when compared to baseline situation, the variation was not statistically significant.

**Figure 3A:**
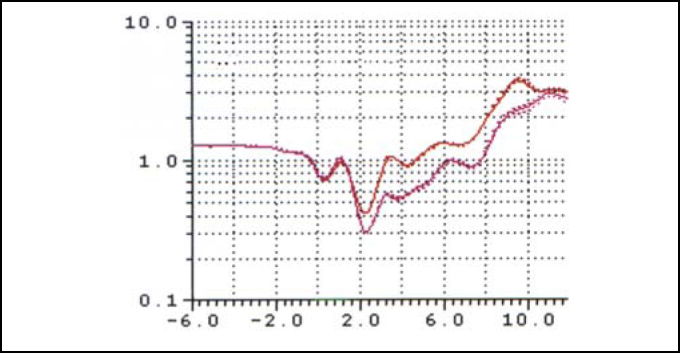
Rhinograms of a typical case (subject n. 6): 1. Baseline conditions (red); 2. with occlusion of nasal valve (pink); 3. after use of nasal decongestant without occlusion of nasal valve (blue).

**Figure 3B:**
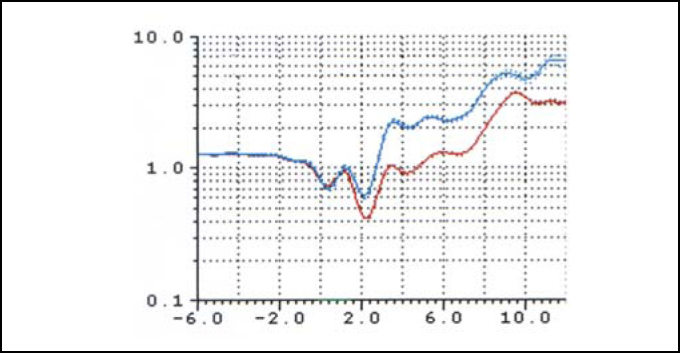
Rhinograms of a typical case (subject n. 6): 1. Baseline condition (red); 2. with occlusion of nasal valve (pink); 3. after use of nasal decongestant without nasal valve occlusion (blue).

**Figure 4:**
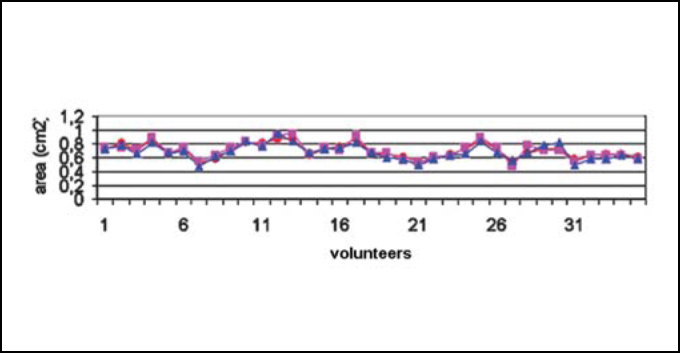
Changes to ASTM1. Baseline conditions (red circles); after obstruction of nasal valve (pink triangle) and after decongestion (blue squares).

**Figure 5:**
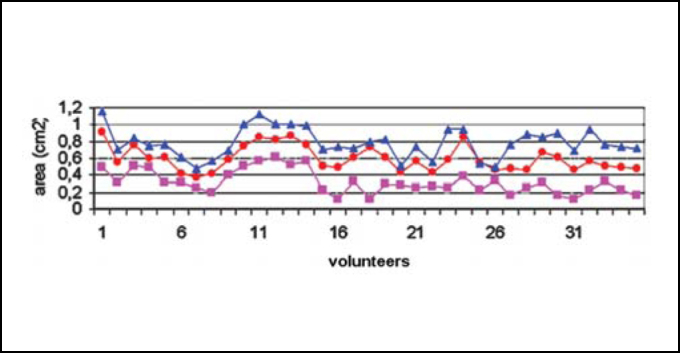
Changes to ASTM2. Baseline conditions (red circles); after obstruction of nasal valve (pink triangles) and after decongestion (blue squares).

**Table 1:** Mean, amplitude and statistical comparison of ASTM1 and ASTM2 at baseline condition, after occlusion of nasal valve and after nasal topical vasoconstrictor.

	baseline condition	NV obstruction	VC after	statistical comparison
MCA 1	0.70 ± 0.10	0.71 ± 0.12	0.68 ± 0.11	ns*
(cm^2^)	0.52 - 0.88	0.47 - 0.94	0.48 - 0.95	ns**
MCA 2	0.60 ± 0.15	0.32 ± 0.14	0.78 ± 0.17	p < 0.001*
(cm^2^)	0.38 - 0.91	0.12 - 0.62	0.48 - 1.15	p < 0.001**

dp: standard deviation VC: nasal topical vasoconstrictor NV: nasal valve Analysis of variance:

^*^ baseline x occlusion NV

^**^ baseline x post-VC

**Table 2:** Means, amplitudes and statistical comparison of DASTM1 and DASTM2 in baseline condition, after occlusion of nasal valve and after nasal topical vasoconstrictor.

	baseline condition	NV obstruction	VC after	statistical comparison
DMCA 1	0.28 ± 0.12	0.28 ± 0.12	0.28 ± 0.12	ns*
(cm)	0.18 - 0.42	0.18 - 0.42	0.18 - 0.42	ns**
DMCA 2	1.93 ± 0.14	2.15 ± 0.25	1.85 ± 0.24	p < 0.001*
(cm)	1.62 - 2.10	1.86 - 2.82	1.38 - 2.86	ns**

dp: standard deviation

VC: nasal topical vasoconstrictor

NV: nasal valve

Analysis of variance:

^*^ baseline x occlusion VN

^**^ baseline x post-VC

The distance from the beginning of the first notch was unaltered in all subjects, the second point before zero cm; thus, there was just one single value and we could not calculate the mean or standard deviation. The distance from the beginning of the notch was the fourth point after zero cm in all subjects; once again, there was just one single value, without mean and standard deviation.

## DISCUSSION

After obstruction of ostium internum, AST1 was not significantly modified. AST2 was significantly reduced and increase after obstruction of ostium internum and decongestion of mucosa, respectively.

In our study, we occluded ostium internum by changing the geometry to observe what would change in the rhinogram. VC, acting over the nasal mucosa, changes the geometry of isthmus nasi because it is comprised by erectile tissue.

Corey et al.^11^ stated that the anatomical correlation of the first notch is not clearly delineated, but it seems to correspond to the region close to NV. They stated that because in their study AST1 was located anteriorly to the distance of NV measured endoscopically. Buenting et al.^18^ reported that the distance of AST, measured by the lowest point of notch in the rhinogram, is consistently overestimated, and there is always a delay in the response for formation of notch. The distance from beginning of obstacle is accurately reflected in the rhinogram as being the most initial point of the notch. Thus, AST1 reported by Corey et al.^11^ can only reflect a narrow region of the anterior nasal cavity to NV.

The initial point of AST1 was always located on the second point before zero cm in all subjects because the adapter used had a tapered format, with adjustable extremity to the tube of widest sound waves than the extremity in contact with the nostril, showing that this notch starts inside the nasal adapter.

AST1 and increase in AST observed right after it did not show modification at rhinogram after use of VC or after obliteration of NV (p = ns), because we did not modify the nostril and the nasal vestibule, despite the posterior location of these structures; thus, this first notch seems to reflect the entry of the nose, or the nostril.

To state that AST1 refers to a structure posterior to the nostril is to say that there is a perfect junction between the nasal adapter and the nostril in all adult Caucasian subjects without nasal affections, or that AR is not capable of detecting narrowing and widening of nostrils that occur in the nasal vestibule exactly in the region in which AR is the most reliable^3^.

Many authors^7^, ^8^, ^9^, ^10^ stated that AST1 referred to NV and AST2 referred to head of inferior nasal concha (CNI). These authors stated that because in their studies, they compared rhinograms before and after VC, or after nasal surgery, and found increase in the area only on the second notch (AST2), AST1 was not modified; thus, they concluded that AST1 was NV and AST2 was the head of CNI. In our studies, after use of VC, we confirmed the same results, because AST2 was significantly changed (p < 0.001); however, when we obstructed the beginning of NV (ostium internum), it was also AST2 that was significantly changed (p < 0.001). Thus, it seems that the second notch is representing the NV as a whole. It is very unlikely that AST1 is representing NV if it remains unaltered after the block of NV.

Some authors^9^, ^18^ conducted studies detecting the changes to ASTM1 and ASTM2 by placing external nasal dilator and after use of VC. They detected mild increase of AST1 and significant increase of AST2 with the use of external nasal dilator; after use of VC, AST1 did not change and AST2 had significant increase, similarly to our study. External nasal dilator acted over the inferior border of the superior lateral cartilage. The confirmation of mild increase in AST1 and significant increase in AST2 with use of external nasal dilator agrees with our study that the second notch is referring to NV as a whole, and not only to the head of CNI, because if it were the case, AST2 would not be changed by the use of external nasal dilator. We believe that mild increase of AST2 is due to the fact that there are fibrous connections interconnecting the caudal margin of the superior alar cartilage with the head margin of inferior alar cartilage as described by Shaida, Kenyon^15^.

After occlusion of NV, the second notch showed significantly reduced AST (p < 0.001) and after application of VC, there was significant increase (p < 0.001). Thus, the second notch should represent narrowing of erectile tissue exactly on the entry of NV, the ostium internum. The distance from the initial part of the notch remained the same, and only the notch area suffered marked reduction, which seemed to be logical because we completely obliterated the orifice.

DAST2 increased after occlusion of NV because since the notch is greater, the descending curve is greater, leading to more posterior location of the lowest point of the notch.

NV is a three-dimension anatomical-functional unit anteriorly comprised by ostium internum and posteriorly by isthmus nasi with 1 to 1.3 cm long^5^, the same length as obstacles used by Buenting et al.^19^ that concluded that AR is not capable of measuring the length of the obstacle. Owing to the fact that the equipment resolution was not good - only 0.35 cm^20^, tracing was unable to provide information on AST affection between ostium internum and isthmus nasi, forming only single notch in the rhinogram.

The literature reported that after use of VC, minimum transversal section area (ASTM) of rhinogram moves anteriorly when it is the second notch^10^, ^20^, ^21^. It is not clear what makes it move anteriorly. It moves anteriorly because:

1) ASTM is located in the first notch^10^, which means that AST in the second notch increases that much as a result of reduction of mucous component of NV, that AST of the first notch is smaller, forming the ASTM of the rhinogram. In this case, we believe that the term anteriorization should not be used, because ASTM starts to represent another notch, another anatomical structure.
2) ASTM continues to be located on the second notch^22^, but this notch is anterior, probably because the notch is smaller; thus, the lowest point of the notch is somewhat more anterior. In this case, two situations may have taken place:
  a) as a result of the reduction of the mucous component of NV present in the isthmus nasi, the ostium internum presents smaller AST than isthmus nasi;
  b) as a result of the reduction of mucous component of NV present in isthmus nasi, AST increases, but its AST continues to be smaller than ostium internum AST.

In situation 2, if there is AST increase with placement of external nasal dilator, it means that it represents ostium internum. If there is none, it means that it represents isthmus nasi. The distance from the notch is not modified, but the beginning of the notch continues to be at the ostium internum.

We observed marked reduction of AST in the second notch, after placement of cotton soaked in Vaseline occluding the whole ostium internum, the opening of NV. We believe that AST was not null owing to the fact that sound waves cross the cotton ball soaked in Vaseline, and we managed to analyze the nasal cavity after this obstacle. The purpose of placing this obstacle is not to completely block sound waves, but rather to interfere in sound waves from a determined and known position, the beginning of NV. In addition, we wanted to assess the point in which there would be modification of tracing at the rhinogram, but we could not clearly identify it.

The purpose of this study was not to determine accuracy of AR in determining ASTM and its distance in studied subjects, but rather to determine what the two notches referred to in the nasal cavity.

Based on the results obtained in our study and in the study by Buenting et al.^19^, we believe that the computer program used to obtain the rhinogram should inform the distances from notches calculated from initial point of notch and not only from the lowest point, as current programs do.

We understand that the first notch starts from the end of the nasal adapter and that the lowest point of the notch represents AST of the nostril. Upon using a tapered nasal adapter, the first notch will start from the next nostril, which is the beginning of the narrowing and its AST represents the nostril. The second notch refers to NV as a whole, starting from ostium internum and its AST represents isthmus nasi. In between both notches, we can probably see the representation of the nasal vestibule in the rhinogram.

It is important to adapt the terminology of anatomical structures to the nasal cavity used by different authors to better understand the description of studies.

In the literature, there are marked differences concerning distances from AST1 and 2 that can be explained by differences in length of nasal adapters or resolution of rhinometry system used that may present higher delays of response for the formation of notches.

Upon studying the rhinogram at baseline, with obliteration of NV, and after decongestion of mucosa, we obtained data that may contribute to anatomical correlation of these two notches. Thanks to correct understanding of measuring in the rhinogram of Caucasian adults without nasal affections, we can compare studies carried out by different authors, study rhinogram of subjects of other ethnic groups and, based on history and anterior rhinoscopy, more efficiently and clearly interpret rhinograms of patients with nasal fossa affections.

## CONCLUSION

The analysis of results suggested that the first notch of rhinogram refers to the nostril and the second one to the nasal valve as a whole.
